# Development and Validation of a Self-Quantification Scale for Patients With Hypertension

**DOI:** 10.3389/fpubh.2022.849859

**Published:** 2022-05-02

**Authors:** Guiyue Ma, Zhuqing Zhong, Yinglong Duan, Zhiying Shen, Ning Qin, Dehua Hu

**Affiliations:** ^1^Third Xiangya Hospital, Central South University, Changsha, China; ^2^Xiangya School of Nursing, Central South University, Changsha, China; ^3^College of Nursing, Anhui University of Traditional Chinese Medicine, Hefei, China; ^4^Key Laboratory of Medical Information Research, Central South University, College of Hunan Province, Changsha, China; ^5^Xiangya School of Life Science, Central South University, Changsha, China

**Keywords:** hypertension, self-quantification, scale, reliability, validity

## Abstract

**Background:**

The self-management ability of patients with hypertension is poor, and self-quantification increases gradually with the development of electronics. Self-quantification for patients with hypertension has important implications for individual health. However, there is a lack of relevant scales at present, and we aim to develop a self-quantified scale for patients with hypertension.

**Methods:**

The instrument was developed based on protection motivation theory with literature review, a qualitative interview study and focus group discussions, and pilot testing. A total of 360 patients with hypertension were investigated using the scale. The psychometric properties of the scale were evaluated concerning validity and reliability employing internal consistency reliability, split-half reliability, test-retest reliability, content validity (S-CVI/Ave and I-CVI), and construct validity (exploratory factor analysis and confirmatory factor analysis).

**Results:**

The final scale had 30 items with seven sub-domains. The Cronbach's α for all domains was 0.900 with a range of 0.817–0.938. The split-half reliability coefficient for all domains was 0.743 with a range of 0.700–0.888. The test-retest reliability coefficient for all domains was 0.880 with a range of 0.849–0.943. The S-CVI/Ave for all domains was 0.922 with a range of 0.906- 0.950, and the I-CVI of each item was a range of 0.800–1.000. The result of confirmatory factor analysis of this scale showed that χ2/df was 2.499, RMSEA = 0.065, GFI=0.865, NFI=0.894, IFI=0.934, TLI=0.914, CFI=0.933, RFI=0.865. The Pearson's coefficients between the total scale and every domain were ranging from 0.347 to 0.695, and each domain ranged from 0.130 to 0.481.

**Conclusion:**

The scale has good validity and reliability and can be used as a self-quantification scale for patients with hypertension.

## Introduction

Incidence of hypertension has increased markedly worldwide owing to improvement of people's living standards and the global social economy (1). Essential hypertension is the most common chronic disease in China. The number of patients with cardiovascular diseases is approximately 330 million according to the 2019 China Cardiovascular Disease Health and Disease report. Notably, hypertension is responsible for 245 million cases, ranking the highest compared with other kinds of cardiovascular diseases (2). Prevalence of hypertension is aggravated by increase in the aging population (3). The prevalence of hypertension among Chinese residents aged ≥ 18 years was 27.9% according to a survey on hypertension conducted between 2012 and 2015 in China. Notably, the survey results showed that the hypertension prevalence among residents aged ≥ 75 years was 59.8% (4). Studies report that 803000 cardiovascular events would be alleviated each year and 1.2 million healthy life years would be obtained if all patients with hypertension are treated (5). The awareness rate, treatment rate, and control rate of hypertension are 36.0%, 22.9%, and 5.7% respectively (6). This indicates that patients with hypertension in China lack inadequate management of elevated blood pressure. Therefore, studies should explore effective methods for management of hypertension to improve prevention and treatment of cardiovascular diseases (7).

Hypertension can gradually and permanently damage target organs, leading to life-threatening complications and death. Hypertension requires lifelong medication and lifestyle changes (4). Although various forms of self-management of hypertension have been developed, it is challenging to obtain accurate data on the health of patients with hypertension. Lack of regular self-management of hypertension hinders awareness and behaviors of patients, thus limiting management of the disease (8). Patients can control known and controllable risk factors associated with hypertension through self-quantitation behavior to prevent related complications and improve their health. Patients should be encouraged to implement self-quantification to control progression of hypertension and reduce incidence of complications (9). However, only few studies have explored self-quantitation of patients with hypertension. Currently, no self-quantification scale has been developed for patients with hypertension based on the protective motivation theory. Motivation plays a role in promoting self-quantitation of patients. Therefore, a self-quantification scale for patients with hypertension should be developed to comprehensively analyze patients' self-quantification behavior from the protective motivation theory. Moreover, the psychological mechanism of self-quantification for patients with hypertension should be evaluated. The scale provides a theoretical and practical basis for understanding the current statistics on hypertension. The scale can be used to explore effective, and targeted intervention for patients with hypertension. The scale can form a basis for developing self-quantification promotion strategies for patients with hypertension. Studies should explore strategies to improve the self-management behavior of patients and improve management of blood pressure.

Medical services in China are characterized by large service demand, uneven distribution of medical resources, high proportion of medical individuals, small coverage of medical security, and insufficient government investment. The most effective way to reduce morbidity and ensure health is to actively participate in self-management of hypertension owing to the limitations of medical services in China (10). Self-management of hypertension involves treatment, diet, exercise and change of living habits. Self-quantification has significantly improved through development of wearable devices and is gradually applied to self-management of patients with hypertension (11). Patients with hypertension use technology devices to continuously and non-invasively monitor vital signs and various behavioral information through self-quantification. In addition, self-quantification lays a basis for treatment of diseases and monitoring of disease progress, which reduced the incidence of diseases (12). Studies report that self-quantification reduces blood pressure (13), improves medication adherence (14), and reduces consultation rate without increasing costs (15). Therefore, patients with hypertension can effectively achieve self-management through self-quantification, and engage in participatory health management with health staff.

Advances in technology facilitate easy collection of health data for patients with hypertension. Gary Wolf and Kevin Kelly, editors of Wired magazine, reported the concept of self-quantification for the first time. They defined self-quantification as use of technology and devices to track and explore patient bodies. Therefore, it is possible to track the behavior of a person by collecting and recording their data. Self-quantification is also known as “self-monitoring”, “self-tracking”, “personal analysis” or “life hacker” (16). Currently, there is no clear definition of self-quantification for hypertension. Previous studies report that self-quantification for hypertension refers to use of technology and devices to record, track and quantify hypertension-related indicators, and to conduct self-adjustment and management through data feedback.

Self-quantification for patients with hypertension is important for improving individual health, but it is characterized by limited application. Motivation is a dynamic intrinsic psychological process or intrinsic dynamical whereby an individual activity is guided, stimulated, and maintained by a goal and the behavior transforms to the goal (17). Studies report that motivation improves good self-management behaviors such as exercise, diet, and adherence to medication in patients with hypertension. Motivation guides, stimulates and promotes self-quantification management behavior of patients with hypertension, which significantly improves disease management in patients with hypertension. Motivation can drive self-quantification behavior, thus strengthening patients' cognition of the disease, enhancing patients' awareness of self-management, improving attention to the disease. Moreover, it improves the cognitive level of disease-related knowledge in patients. This study adopted the protection motivation theory (PMT), which comprises three parts namely; information source, cognitive intermediary, and coping model (18). The model is mainly used for prediction of health behavior and health intervention, to explore emergence of health-related behaviors from the perspective of motivational factors.

Although self-quantification for patients with hypertension is a form of management focusing on health, self-efficacy, and pursuit of achievement, previous studies have not fully explored self-quantization and the associated factors and motivations. Therefore, patients with hypertension should be evaluated to reveal their behavior. Currently, there is no psychometric self-quantification scale for patients with hypertension. The aim of the study was to develop and validate a scale of self-quantification scale for patients with hypertension. This scale can be used to evaluate self-quantification of patients with hypertension.

## Methods

### Development of the Self-Quantification Scale for Patients With Hypertension

Previous and guidelines from various international hypertension associations were reviewed to provide a basis for development of the scale (19–21). Patients with hypertension above 18 years old, had no communication barrier and agreed to participate in the study voluntarily, and signed the informed consent form were interviewed. Data saturation was used as the termination index in the interview. A total of 15 subjects were included in the study. The content of the interview was based on protective motivation theory (PMT). The factors that affected self-quantification of patients were recorded and the relevant items were extracted using the semi-structured interview. The number of semi-structured interviews was saturated. The interview was stopped after getting the relevant data. The programmed decision method was used for item selection. A focus group with 7 individuals was formed to conduct item selection. The focus group comprised one chief nurse, two supervisor nurses, one nurse, and three postgraduate students. The focus group discussed and confirmed the structure of the instrument based on PMT. The instrument had seven domains including external reward, internal reward, severity, susceptibility, response efficacy, self-efficacy, and response cost. The group proposed some possible items under each of the facets within each domain, resulting in a pool of 43 items, after reviewing self-quantification motivations instruments (21) and considering the elements of Chinese culture. A convenience sample of ten patients with hypertension was used to obtain feedback on the language and clarity of the questionnaire.

Further, 15 experts were asked to score the relevance and importance of each item on a scale of 1 (extremely irrelevant) to 4 (extremely relevant) and 1 (extremely unimportant) to 5 (extremely important). In addition, the experts modified and/or increased or decreased each item according to their professional knowledge and work experience on the scale. The preliminary scale was modified to comprise 37 items after two rounds of Delphi surveys. A pre-test was carried out at the hypertension ward, outpatient clinic of a tertiary hospital, and one community in Changsha, China from October to November 2021 using the preliminary scale. A total of 185 respondents filled the questionnaires. Various statistical analyses (coefficient of variation, correlation analysis, project differentiation analysis, Cronbach coefficient, and factor analysis) were used to re-screen the items based on the pre-test data. Ultimately, 30 items were selected to form the scale, which comprised seven domains.

### Evaluation of the Scale

#### Process of Evaluating the Scale

A survey was carried out at the ward and outpatient clinic of a tertiary hospital, two community hospitals, and five residential areas in Changsha, China from November to December 2021. The inclusion criteria were as follows: (1) patients with hypertension over 18 years old; (2) patient able to read and understand the questionnaires; (3) patients who signed the informed consent form. The exclusion criteria were: (1) patients with hypertensive crisis, hypertensive encephalopathy, or acute hypertension; (2) patients presenting with other major diseases, such as advanced malignant tumors. The sample size should be 5 to 10 times the number of items (22), and considering that individuals may be lost during the retest, the sample size was increased by 20%. Therefore, a total of 360 individuals were included in the study. Investigators sent questionnaires to patients with hypertension who met the inclusion criteria. Participants were briefed on the anonymity and confidentiality of the survey. In addition, the subjects were informed on the purpose and significance of the survey, and were asked to sign an informed consent form. Answers were checked immediately after submission of the questionnaire by the investigators to ensure their integrity. The questionnaire was returned to the patients, incase of missing values, for the participants to complete the missed items. The study was approved by the Ethics Committee of Central South University (certification number: E202071) and all participants signed informed consent before participation. The scale was applied to all participants. Information was obtained during a 10 to 15 min face-to-face conversation.

#### Validity of the Scale

The raw scores of items, domains, and overall scale were calculated based on the data collected. Each item of the scale was rated on a five-level Likert scoring system, namely, totally disagree, disagree, uncertainty, agree, totally agree. Positively stated items were scored from 1 to 5, whereas items 31, 33, 34 were reversely scored. Each domain score was obtained by computing the sum of the within-domain item scores. The overall scale score was the sum of the seven domain scores. The scale scores ranged from 30–150 points. A high total table score indicated high self-quantification. Validity of the scale was evaluated by content validity, exploratory factor analysis (EFA), and confirmatory factor analysis (CFA). Content validity was calculated according to the results of the second round of Delphi surveys, including the I-CVI of each item level, the S-CVI between each dimension and the scale, and the S-CVI/Ave of each dimension and scale. The evaluation threshold for content validity was I-CVI ≥ 0.78. A Kappa value K^*^ > 0.74, obtained after correcting the random consistency of experts, indicated that the content validity of the item was good, and S-CVI/Ave ≥ 0.90 indicated that the content validity of each dimension and the whole scale was good. EFA was used to determine whether it was suitable for factor analysis according to the calculated results of sampling appropriateness using Kaiser-Meyer-Olkin (KMO) and Bartlett's Test of Sphericity. The KMO value was between 0 and 1, KMO > 0.7 and Bartlett's spherical test results indicate that the results are significant (*P* < 0.05), implying that factor analysis was suitable. The maximum variance method of Principal Component Analysis and Orthogonal Rotation was used to select common factors based on the theoretical extraction of 7 fixed factors. An item was deleted when the item factor load was less than 0.4, otherwise, whereas a item factor load above 0.4 indicated that the scale had good structural validity. CFA was used to verify the subject based on the outcome of the EFA. The model fitting index comprised X^2^/DF, GFI, RMSEA, NFI, IFI, TLI, CFI and RFI. A X^2^/DF < 3 indicated good fitness, RMSEA < 0.08 and a smaller value implied that the model has fitting. A value less than 0.05 indicated that the model had a good fitting a range between 0.05 and 0.08 implied that the model fits well. Values above 0.9 and more than 0.85 for the other indexes were acceptable. In this study, CFA was used to evaluate the convergent validity of the scale in each dimension. Good convergent validity is indicated by: Standardized Factor Loadings > 0.45, a Composition Reliability > 0.7, and Average Variance Extracted >0.5. Discriminant validity was calculated through correlation analysis of the different dimensions. A low correlation coefficient between different dimensions of the scale indicates high discriminant validity.

#### Reliability

**Figure 1:**
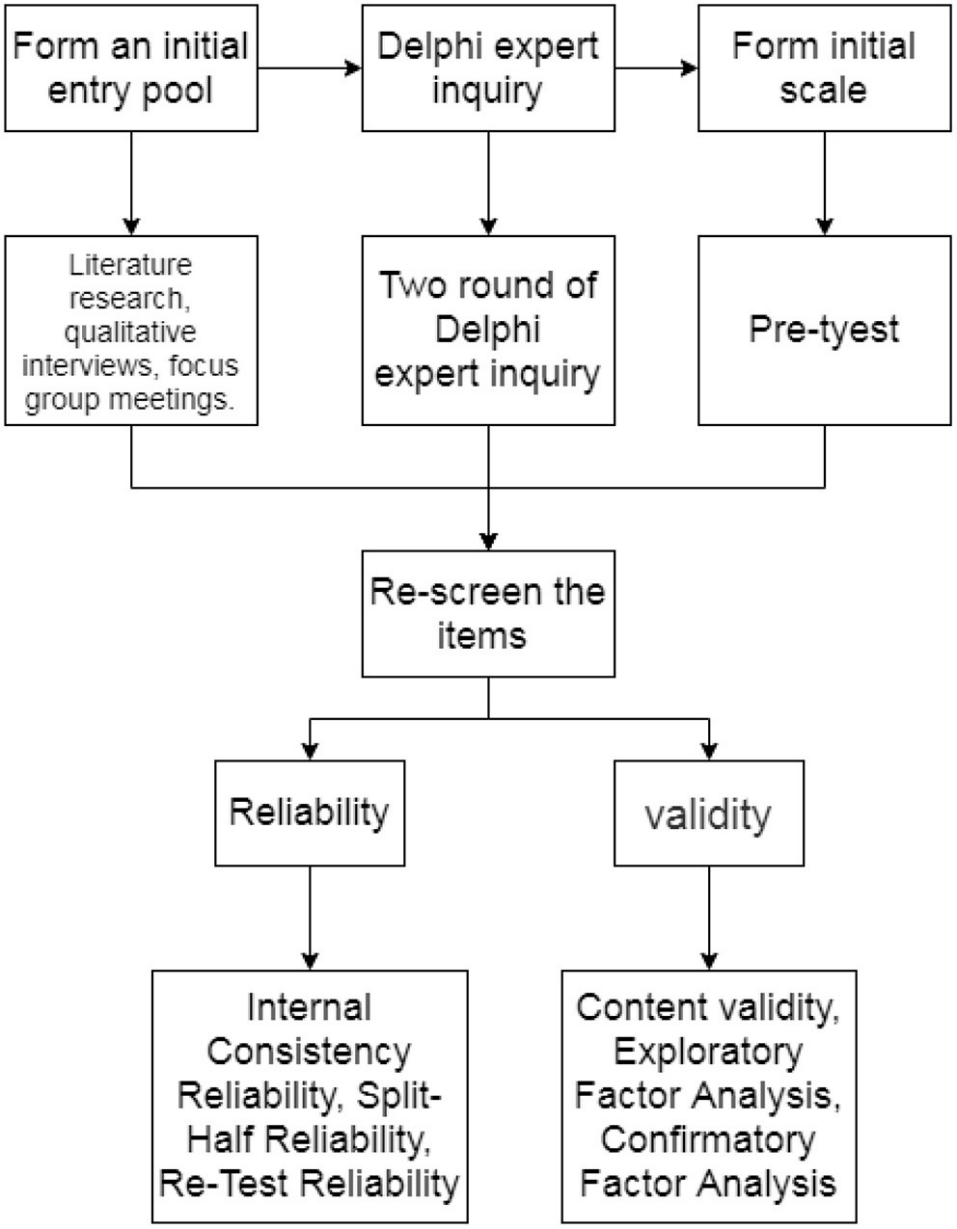
The study design.

Internal consistency was evaluated using the Cronbach's α for each domain. Half-reliability of the two parts was calculated using the Spearman-brown formula. Subsequently, 30 randomly selected hypertension patients were examined with the self-quantification scale after every 2 weeks to determine the test-retest reliability. The study design is presented in Figure 1.

## Results

### Pre-test Results

#### Socio-Demographic and Clinical Characteristics of Participants

Individuals aged between 50 and 60 account for the largest proportion (33.0%) of patients with hypertension included in the present study. Out of the 180 included subjects, 54.1% were male and 156 (84.3%) were married. The level if education of most subjects was primary school and below accounting for 34.6% of the participants (Table 1).

**Table 1:** Socio-demographic and clinical characteristics of the participants (*N* = 185).

**Variables**	**Number**	**Percentage (%)**
Gender		
Male	100	54.1
Female	85	45.9
Age group(years)		
18~30	14	7.57
31~40	8	4.32
41~50	11	5.95
51~60	61	33.0
61~70	47	25.4
≥70	44	23.8
Marriage		
Unmarried	6	3.2
Married	156	84.3
Bereaved a spouse	23	12.4
Education level		
Primary school and below	64	34.6
Junior middle school	60	32.4
High school	27	14.6
Bachelor degree or above	34	18.4
Occupation		
Be unemployed	15	8.1
Farming	19	10.3
On the job	51	27.5
Retire	100	54.1
Monthly per capita household income		
<1000	54	29.2
1000~3000	59	31.9
3001~5000	40	21.6
>5000	32	17.3
Home place		
City	149	80.5
Rural areas	36	19.5
Housing		
Live alone	18	9.7
Live with family	167	90.3
Medicare type		
Medical insurance for urban residents	87	47.0
The new type of rural cooperative medical care	96	51.9
Own expense	2	1.1
Disease course(years)		
<1	26	14.1
1~5	29	15.7
6~10	38	20.5
>10	92	49.7
Complications		
No	97	52.4
Yes	88	47.6
Type of medication		
1	157	84.8
2	19	10.3
≥3	9	4.9
Family history		
No	94	50.8
Yes	91	49.2

#### Scale-Entry Quality Analysis and Screening Results

A total of 200 questionnaires were issued in the pre-test. Notably, 185 valid questionnaires were recovered, with an effective recovery rate of 92.5%.

##### Results From Coefficient of Variation Analysis

The results showed that items 10, 13, 14, 20, 22, 23, 26, and 28 had a CV <25%, thus they were deleted (Table 2).

**Table 2:** Mean, standard deviation, and coefficient of variation of each item (*N* = 185).

**Item**	**Mean**	**SD**	**CV(%)**	**Item**	**Mean**	**SD**	**CV(%)**
1	2.92	1.37	47.10	20	4.10	0.64	15.50^*^
2	2.95	1.19	40.46	21	2.21	1.52	68.56
3	3.17	1.31	41.20	22	4.04	0.74	18.38^*^
4	3.12	1.47	47.12	23	3.98	0.80	20.18^*^
5	3.89	1.07	27.66	24	3.71	1.16	31.25
6	3.58	1.07	29.88	25	3.18	1.47	46.06
7	3.76	1.02	27.05	26	4.00	0.98	24.59^*^
8	3.43	1.26	36.79	27	3.76	1.01	26.91
9	3.87	1.08	27.92	28	3.94	0.95	23.98^*^
10	4.05	0.97	23.81^*^	29	3.86	0.97	25.13
11	3.78	1.03	27.31	30	4.00	1.02	25.40
12	3.76	1.00	26.62	31	2.63	1.56	59.34
13	3.97	0.93	23.34^*^	32	2.62	1.47	56.21
14	4.02	0.84	21.01^*^	33	2.97	1.40	47.25
15	3.12	1.23	39.49	34	1.86	1.29	69.39
16	2.62	1.12	42.90	35	3.68	1.18	31.93
17	2.60	1.19	45.58	36	3.37	1.28	37.81
18	2.41	1.10	45.64	37	3.21	1.31	40.77
19	2.82	1.18	41.86				

* *The bold values indicates CV <25%*.

##### Item Differentiation Degree Analysis Results

Independent sample t-test were used to test the difference between low score group (97.62 ± 9.90) and high score group (151.14 ± 12.85) classified according to the total score of self-quantified motivation of hypertension patients. Only item 37 had a P-value >0.05 indicating that there was no significant difference between the high and low groups, therefore, this item was removed (Table 3).

**Table 3:** Test of differences between low and high groups (*N* = 185).

**Item**	**t**	**p**	**Item**	**t**	**p**	**Item**	**t**	**p**
1	−7.415	0.000	14	−5.368	0.000	27	−3.168	0.002
2	−6.398	0.000	15	−14.277	0.000	28	−5.319	0.000
3	−5.741	0.000	16	−14.526	0.000	29	−3.889	0.000
4	−15.525	0.000	17	−12.234	0.000	30	−5.080	0.000
5	−3.785	0.000	18	−8.059	0.000	31	−8.126	0.000
6	−3.888	0.000	19	−12.035	0.000	32	−2.559	0.014
7	−7.648	0.000	20	−4.722	0.000	33	−7.452	0.000
8	−6.258	0.000	21	−12.509	0.000	34	−6.221	0.000
9	−3.687	0.000	22	−5.070	0.000	35	2.459	0.016
10	−6.487	0.000	23	−5.875	0.000	36	2.448	0.016
11	−5.836	0.000	24	−9.195	0.000	37	−0.083	0.934^*^
12	−6.415	0.000	25	−9.195	0.000			
13	−6.267	0.000	26	−5.087	0.000			

* *The bold values indicates P > 0.05*.

##### Correlation Coefficient Analysis Results

Correlation analysis was conducted and items 27, 32, 35, 36, and 37 had a correlation coefficient < 0.3, implying that the entries were less representative thus they were removed (Table 4).

**Table 4:** Relationship between entry and total score of each dimension and total score of the scale (*N* = 185).

**Item**	**Score of each dimension**	**Total score of the scale**	**Item**	**Score of each dimension**	**Total score of the scale**
1	0.856^**^	0.457^**^	20	0.622^**^	0.472^**^
2	0.716^**^	0.422^**^	21	0.724^**^	0.729^**^
3	0.799^**^	0.417^**^	22	0.667^**^	0.477^**^
4	0.767^**^	0.679^**^	23	0.690^**^	0.479^**^
5	0.704^**^	0.320^**^	24	0.804^**^	0.554^**^
6	0.708^**^	0.314^**^	25	0.800^**^	0.533^**^
7	0.414^**^	0.525^**^	26	0.901^**^	0.391^**^
8	0.696^**^	0.349^**^	27	0.882^**^	0.263^**^
9	0.784^**^	0.311^**^	28	0.937^**^	0.393^**^
10	0.620^**^	0.536^**^	29	0.900^**^	0.328^**^
11	0.805^**^	0.486^**^	30	0.863^**^	0.337^**^
12	0.802^**^	0.510^**^	31	0.569^**^	0.478^**^
13	0.670^**^	0.428^**^	32	0.627^**^	0.241^**^
14	0.861^**^	0.484^**^	33	0.508^**^	0.485^**^
15	0.832^**^	0.587^**^	34	0.642^**^	0.469^**^
16	0.859^**^	0.642^**^	35	0.319^**^	0.173^*^
17	0.774^**^	0.647^**^	36	0.374^**^	**0.113**
18	0.797^**^	0.488^**^	37	0.491^**^	**0.107**
19	0.770^**^	0.543^**^			

** *indicates significant correlation at 0.01 (two-sided)*;

* *significant correlation at 0.05 (two-sided). The bold values indicates correlation coefficient < 0.3*.

##### Cronbach's Coefficient Analysis Results

Cronbach's coefficient for items 32, 35, 36, and 37 increased compared with the original scale, therefore, these items were eliminated (Table 5).

**Table 5:** Cronbach's coefficient and change of scale (*N* = 185).

**Item**	**The Cronbach's α value of the deleted item**	**The Cronbach's value changes**	**Item**	**The Cronbach's α value of the deleted item**	**The Cronbach's value changes**
1	0.882	↓	20	0.882	↓
2	0.881	↓	21	0.874	↓
3	0.882	↓	22	0.881	↓
4	0.875	↓	23	0.881	↓
5	0.881	↓	24	0.878	↓
6	0.882	↓	25	0.879	↓
7	0.880	↓	26	0.879	↓
8	0.883	↓	27	0.881	↓
9	0.881	↓	28	0.879	↓
10	0.880	↓	29	0.880	↓
11	0.880	↓	30	0.880	↓
12	0.880	↓	31	0.881	↓
13	0.880	↓	32	0.885	**↑**
14	0.880	↓	33	0.881	↓
15	0.877	↓	34	0.880	↓
16	0.877	↓	35	0.891	**↑**
17	0.877	↓	36	0.891	**↑**
18	0.880	↓	37	0.887	**↑**
19	0.878	↓			

##### Exploratory Factor Analysis Results

The results showed that the factor load of items 10, 35, and 36 was less than 0.4, item 2 was present in 2 factors, and items 7 and 21 were not present in their dimension. The results indicate that these three items were not representative, and the above entries were deleted. Seven items that did not meet the criteria were eliminated from the formal survey version of the self-quantified motivation scale for hypertension patients. The final version comprised 7 dimensions and a total of 30 items.

### Reliability and Validity of the Scale

#### Socio-Demographic and Clinical Characteristics of the Participants

Participants between the ages of 60 and 70 accounted for the largest proportion (43.3%) of the hypertension patients included in the test. Out of the 360 participants included in the validity test, 56.4% were female and 317 (88.1%) married. Most of the participants were in the junior high school education level group, accounting for 38.9% of all participants. Undergraduate students and higher levels accounted for a low proportion (10.0%) of participants. The results showed that 213 (59.2%) participants were retired. The monthly per capita household income of 133 (36.9%) patients with hypertension ranged between 1000 and 3000. Notably, 306 (85.0%) patients with hypertension lived in cities, and most of the participants lived with their families (93.1%). The findings showed that 179 (49.7%) patients with hypertension were insured by urban residents, and most of them had presented with hypertension for more than 10 years (56.1%). Notably, 56.2% of the subjects had complications, most of them took one antihypertensive drug (84.4%) and had a family history (56.1%) of hypertension (Table 6).

**Table 6:** Sociodemographic and clinical characteristics of participants (*N* = 360).

**Variables**	**Number**	**Percentage (%)**
Gender		
Male	157	43.6
Female	203	56.4
Age group(years)		
18~30	3	0.8
31~40	7	1.9
41~50	41	11.4
51~60	96	26.7
61~70	156	43.3
≥70	57	15.8
Marriage		
Unmarried	2	0.6
Married	317	88.1
Bereaved a spouse	33	9.2
Divorce	8	2.2
Education level		
Primary school and below	121	33.6
Junior middle school	140	38.9
High school	63	17.5
Bachelor degree or above	36	10.0
Occupation		
Be unemployed	17	4.7
Farming	8	2.2
On the job	122	33.9
Retire	213	59.2
Monthly per capita household income		
<1000	116	32.2
1000~3000	133	36.9
3001~5000	91	25.3
>5000	20	5.6
Home place		
City	306	85.0
Rural areas	54	15.0
Housing		
Live alone	25	6.9
Live with family	335	93.1
Medicare type		
Medical insurance for urban residents	179	49.7
The new type of rural cooperative medical care	169	46.9
Own expense	12	3.3
Disease course(years)		
<1	6	1.7
1~5	73	20.3
6~10	79	21.9
>10	202	56.1
Complications		
No	158	43.9
Yes	202	56.2
Type of medication		
1	304	84.4
2	47	13.1
≥3	9	2.5
Family history		
No	158	43.9
Yes	202	56.1

#### Validity

##### Content Validity

The results showed that the I-CVI range of each item in the formal scale was 0.800-1.000, and all items had I-CVI value above 0.78. The S-CVI/Ave of the total table was 0.922. The average S-CVI of the different dimensions were 0.910, 0.906, 0.950, 0.932, 0.906, 0.920, and 0.933, which were all above 0.90. The K^*^ value of all entries was greater than 0.74, indicating that the scale was reliable.

##### Exploratory Factor Analysis

The Kaiser-Meyer-Olkin score of sampling adequacy was 0.78. Bartlett's test of sphericity was significant (χ2=7368.710, df =435, *P* < 0.001). Seven sub-domains contributed to 71.57% of the total variance. Factor analysis results for the scale are shown in Table 7.

**Table 7:** Results of factor analysis for the scale.

**Sub-dimensions Item number**	**Item**	**Factor loading**	**Variation explained (%)**
External Reward			13.83
1	Most of my friends and relatives measure their blood pressure regularly.	0.88	
2	Most of my friends and relatives approve of my using smart devices to measure blood pressure.	0.86	
3	My friends and relatives encourage me to use smart devices to measure blood pressure.	0.63	
Internal Reward			25.33
4	The regular measurement of blood pressure by an intelligent device is helpful for me to know the changes in blood pressure in time.	0.71	
5	Monitoring heart rate with an intelligent device is helpful for me to adjust the amount of exercise in time.	0.81	
6	Limiting daily salt intake according to the recommendations of intelligent devices is good for the stability of blood pressure.	0.65	
7	Regular weight measurement with intelligent devices is helpful for me to adjust my eating habits.	0.75	
8	Use an intelligent device to record in time after taking antihypertensive medication to avoid misuse.	0.83	
Severity			36.31
9	Patients with insufficient exercise or excessive hypertension are not conducive to blood pressure stability.	0.76	
10	Patients with hypertension who have a salty diet that is not conducive to blood pressure stability.	0.78	
11	Patients with hypertension who are too obese are not conducive to blood pressure stability.	0.68	
12	Patients with hypertension do not insist on taking antihypertensive medication is not conducive to blood pressure stability.	0.87	
Susceptibility			46.34
13	If it does not adhere to the regular measurement of blood pressure, it is difficult to find abnormal blood pressure in time.	0.69	
14	Does not monitor the heart rate in real-time, it is difficult to adjust the amount of exercise in time.	0.83	
15	Does not know the calorific value of food, it is difficult to develop light eating habits.	0.79	
16	If the weight is measured irregularly, it is difficult to control the weight accurately.	0.83	
17	Does not have regular medication reminders, it is difficult to take medicine on time and regularly.	0.59	
Response Efficacy			55.77
18	I can manage blood pressure reasonably by measuring blood pressure regularly through intelligent devices.	0.80	
19	The ranking of steps displayed by smart devices can inspire me to increase the amount of exercise.	0.81	
20	Diet plans provided through smart software (for example, oil and salt intake) can promote blood pressure stability.	0.81	
21	I can inspire myself to control my weight through weight management with intelligent software.	0.81	
22	The regular medication reminder function provided by the intelligent device can urge me to take antihypertensive drugs on time.	0.58	
Self-Efficacy			63.68
23	I am confident that I will continue to use an intelligent sphygmomanometer or other devices to monitor blood pressure.	0.87	
24	I am confident that I will continue to use a device that monitors heart rate or exercise volume for exercise management.	0.90	
25	I am confident that I will eat reasonably according to the type and quantity of food recommended by the intelligent software.	0.91	
26	I am confident that I can control my weight according to the diet plan recommended by the intelligent software.	0.89	
27	I am confident that I will take antihypertensive medication correctly according to the instructions of intelligent software.	0.84	
Response Cost			71.57
28	I was worried about the abnormal blood pressure values and body weight measured by the smart device.	0.84	
29	It may not accurately measure blood pressure, blood pressure, and oxygen saturation through intelligent devices, affecting the judgment of the disease.	0.83	
30	Regular measurement of blood pressure, amount of exercise, and weight will take up my time for work, entertainment and leisure.	0.81	

##### Confirmatory Factor Analysis

CFA calculation of the 360 data of the formal survey showed that the χ2/df was 2.499, which met the cut-off of <3. The RMSEA was 0.065, which is less than 0.08. IFI, TLI and CFI were above 0.9, and d GFI, NFI and RFI were above 0.85, which meet the acceptable range threshold. These findings show that the model fitting metrics of the scale were within the acceptable range, indicating that the scale had good model adaptation. The AVE values of the different dimensions were 0.633, 0.560, 0.600, 0.559, 0.605, 0.775, and 0.698. All AVE values were >0.5. The CR values of the various dimensions were 0.835, 0.862, 0.856, 0.863, 0.882, 0.945, and 0.874. All CR values were above 0.7, thus they met the standard of convergence validity. Correlation coefficients between the total score of all domains and scales were above 0.5, indicating that the total score of each dimension and scale was above moderate, and the correlation was significant (*P* < 0.01). The absolute values of correlation coefficients between each sub-table ranged between 0.130 and 0.481. The values reached significant levels indicating a consistent direction of each sub-table. The absolute values of the correlation coefficients between each subscale and the total table ranged between 0.548 and 0.740 and reached a significant level. This implies that the correlation was above-biased (Table 8).

**Table 8:** Correlation analysis of the scale.

	**External reward**	**Internal reward**	**Severity**	**Susceptibility**	**Response efficacy**	**Self-efficacy**	**Response cost**	**Total scale**
Internal reward	0.236							
Severity	0.264	0.282						
Susceptibility	0.470	0.245	0.326					
Response efficacy	0.262	0.254	0.257	0.334				
Self-efficacy	0.301	0.171	0.130	0.235	0.163			
Response cost	0.387	0.163	0.364	0.481	0.286	0.214		
Total scale	0.675	0.559	0.570	0.740	0.581	0.548	0.629	1.000

#### Reliability of the Scale

Reliability of the scale was evaluated using three procedures namely: internal consistency, test-retest, and split-half reliability (Table 9). The Cronbach's α for the seven domains were above 0.80. The total Cronbach's α of the scale was 0.90, and the Cronbach's α for the domains ranged between 0.82 and 0.94. The total split-half reliability of the scale was 0.74, and that from the domains ranged between 0.70 and 0.89. The test-retest correlation coefficients (r) for the seven domains ranged between 0.85 and 0.94, with *r* = 0.88 for the overall scale. Differences in domain scores between the first and the second assessments were not statistically significant for most of the domains (p > 0.05).

**Table 9:** Reliability coefficients of the total scale and each domain (*N* = 360).

**Domain**	**Item**	**Cronbach's α**	**Split-Half Reliability**	**Test-Retest**
External Reward	3	0.839	0.700	0.868
Internal Reward	5	0.826	0.819	0.892
Severity	4	0.823	0.794	0.849
Susceptibility	5	0.869	0.798	0.883
Response Efficacy	5	0.817	0.742	0.903
Self-Efficacy	5	0.938	0.888	0.943
Response Cost	3	0.924	0.799	0.880
Total Scale	30	0.900	0.743	0.880

## Discussion

### Verification of the Self-Quantification Scale for Patients With Hypertension

Instruments must be validated based on at least two aspects: validity and reliability. Validity is the extent of accuracy by which the instrument measures the target issue. A scale instrument was established following programmed decision procedures for patients with hypertension. The instrument was developed by various methods including group discussion, interview, Delphi inquiry method. A pre-test was conducted to effectively reduce the number of items in the final version to 30 items from an initial 43-item pool. This process ensures good content validity and sound conceptual structure of the instrument. Content validity, EFA, and CFA were used to evaluate the construct and criterion-related validity of the scale. Correlation analyses showed a strong correlation between domains and the total scale, but weak correlations between items and other domains. Factor analysis revealed that the components extracted from the data were consistent with the theoretical constructs of the instrument, confirming the construct validity of the scale.

Reliability refers to reproducibility or consistency of item scores from one assessment to another. Internal consistency reliability (Cronbach's α), split-half reliability, and test-retest reliability were used to explore reliability of the instrument in the current study. The results showed that the internal consistency coefficients of the scale domains as well as the overall score were >0.80. The split-half reliability of the scale domains as well as the overall score was >0.70. Notably, the test-retest reliability coefficient for the overall score was 0.88, whereas that of the individual domain scores were all >0.80. These findings indicate that the instrument has excellent reliability.

### Application of the Scale

The present study provides a reliable evaluation tool for self-examination to understand the psychological needs and tendency of patients with hypertension. The tool provides a basis for medical staff to understand issues of patients with hypertension. This will enable formulation of personalized and effective intervention measures, thus improving hypertension management and blood pressure control strategies. The theoretical framework of the scale was PMT, which provides a research basis for exploring the factors that affect self-quantification of patients and helps in improving the self-quantification level in the future. The scale provides a basis for stimulating self-quantification enthusiasm of patients and promoting self-quantification among patients with hypertension.

## Study Limitations

The sample size of the study was small, which may affect validity of the findings. Follow-up large cross-sectional studies should be conducted to explore validity of the self-quantitation scale for patients with hypertension developed in this study. In addition, participants were derived from one urban area, therefore, a multicenter survey should be conducted to verify the findings. An intervention index system of self-quantitation management for patients with hypertension should be constructed based on the results of a large sample investigation and analysis. Moreover, a set of scientific, comprehensive, and feasible intervention strategies should be formulated to improve the management of hypertension in our country.

## Conclusion

The findings showed that the scale developed in the present study had good psychometric properties. Therefore, it is a useful instrument for self-quantitation of patients with hypertension in China. The instrument and future versions have the potential to be implemented in other countries to improve self-quantitation of patients with hypertension.

## Data Availability Statement

The original contributions presented in the study are included in the article/Supplementary Material, further inquiries can be directed to the corresponding authors.

## Ethics Statement

The studies involving human participants were reviewed and approved by Research Ethics Board of Third Xiangya of Central South University. The patients/participants provided their written informed consent to participate in this study.

## Author Contributions

GM designed the study, participated in the data processing and statistical analysis, and wrote the initial draft of the manuscript. YD participated in the design of the study and questionnaire administration and discussed analytical results. ZS and NQ provided important feedback on the manuscript. ZZ and DH participated in its design and provided important feedback on the manuscript. All authors read and approved the final manuscript.

## Funding

This study was supported by a grant from the Special Science Popularization Project for the Construction of innovative provinces in 2020-2021(Grant No.2021zk419).

## Conflict of Interest

The authors declare that the research was conducted in the absence of any commercial or financial relationships that could be construed as a potential conflict of interest.

## Publisher's Note

All claims expressed in this article are solely those of the authors and do not necessarily represent those of their affiliated organizations, or those of the publisher, the editors and the reviewers. Any product that may be evaluated in this article, or claim that may be made by its manufacturer, is not guaranteed or endorsed by the publisher.
