# Supplementary material for: Development and Validation of a Self-Quantification Scale for Patients With Hypertension
Source: Front Public Health. 2022 May 2;10:849859. doi: 10.3389/fpubh.2022.849859 (PMC9132107; doi:10.3389/fpubh.2022.849859)
Supplement: Supplementary file 1 [file Data_Sheet_1.docx]

The results of the extracted information of the interview

**Threat assessment:**

(1)External reward refers to the externally perceived benefits of self-quantification in patients with hypertension.

People with hypertension said that most of their friends and relatives measure their blood pressure regularly; most of their relatives and friends approve of their use of intelligent equipment to measure blood pressure; their friends and relatives encouraged them to use intelligent equipment to measure blood pressure; affected by external factors such as relatives and friends, patients voluntarily use self-quantification to manage blood pressure.

(2)Internal reward refers to the self-perceived benefits of self-quantification in patients with hypertension.

Patients with hypertension said that intelligent equipment that measures blood pressure regularly helps to keep abreast of changes in blood pressure; monitoring heart rate with intelligent equipment is beneficial to adjust the amount of exercise in time; limiting daily salt intake according to the recommendations of intelligent equipment is good for blood pressure stability; intelligent equipment measure weight regularly to help to adjust your eating habits; after taking antihypertensive drugs, use intelligent equipment to record in time to avoid misuse.

(3)Severity refers to the judgment of patients with hypertension on the harmfulness of the disease.

Patients with hypertension generally believe that insufficient or excessive exercise is not conducive to the stability of blood pressure; the salty diet of patients with hypertension is not conducive to the stability of blood pressure; obesity in patients with hypertension is not conducive to blood pressure stability; patients with hypertension do not insist on taking antihypertensive drugs is not conducive to blood pressure stability.

(4)Susceptibility refers to the response of patients with hypertension to the harm of the disease that affects their likelihood.

Patients with hypertension think that if they do not measure their blood pressure regularly, it is difficult to find abnormal blood pressure in time; it is difficult to adjust the amount of exercise without real-time monitoring of heart rate.; without knowing the calorific value of food, it is difficult to develop light eating habits; if you measure your weight irregularly, it is difficult to accurately control your weight without regular medication reminders, and it is difficult to take medicine on time.

**Coping assessment**

(1)Response efficacy refers to hypertension patients' understanding of the benefits of self-quantitative management.

Patients with hypertension believe that blood pressure can be reasonably managed by regularly measuring blood pressure through intelligent equipment; the step ranking displayed by intelligent equipment can motivate me to exercise more; the diet plan provided by smart software (for example, the intake of oil and salt) can promote the stability of blood pressure; weight management through smart software can motivate me to control my weight; and the regular medication reminder function provided by intelligent equipment can urge me to take antihypertensive drugs on time.

(2)Self-efficacy refers to the confidence of patients with hypertension in whether they can take self-quantification.

Whether patients with hypertension have confidence in using intelligent sphygmomanometer or other equipment to monitor blood pressure; whether they have confidence in using equipment for monitoring heart rate or amount of exercise for exercise management; whether they have confidence in eating reasonably according to the type and amount of food recommended by intelligent software; whether they have confidence in controlling weight according to the diet plan recommended by intelligent software; whether they have confidence in taking antihypertensive drugs correctly as suggested by intelligent software.

(3)Response cost refers to the difficulty and cost of hypertension patients' participation in self-quantification.

Patients with hypertension think that the abnormal blood pressure values and body weight measured by intelligent equipment make them worried; the values of blood pressure, heart rate, and blood oxygen saturation measured by intelligent equipment may be inaccurate, which may affect the judgment of the disease; adhere to the regular measurement of blood pressure, exercise and weight will take up my time for work, entertainment and leisure.
